# Identification of trans-eQTLs using mediation analysis with multiple mediators

**DOI:** 10.1186/s12859-019-2651-6

**Published:** 2019-03-29

**Authors:** Nayang Shan, Zuoheng Wang, Lin Hou

**Affiliations:** 1 Center for Statistical Science, Tsinghua University, Beijing, 100084 China; 2 Department of Industrial Engineering, Tsinghua University, Beijing, 100084 China; 3 Department of Biostatistics, Yale School of Public Health, New Haven, CT 06510 USA; 4 MOE Key Laboratory of Bioinformatics, School of Life Sciences, Tsinghua University, Beijing, 100084 China

**Keywords:** Trans-eQTL, Mediation analysis, Multiple mediators

## Abstract

**Background:**

Mapping expression quantitative trait loci (eQTLs) has provided insight into gene regulation. Compared to cis-eQTLs, the regulatory mechanisms of trans-eQTLs are less known. Previous studies suggest that trans-eQTLs may regulate expression of remote genes by altering the expression of nearby genes. Trans-association has been studied in the mediation analysis with a single mediator. However, prior applications with one mediator are prone to model misspecification due to correlations between genes. Motivated from the observation that trans-eQTLs are more likely to associate with more than one cis-gene than randomly selected SNPs in the GTEx dataset, we developed a computational method to identify trans-eQTLs that are mediated by multiple mediators.

**Results:**

We proposed two hypothesis tests for testing the total mediation effect (TME) and the component-wise mediation effects (CME), respectively. We demonstrated in simulation studies that the type I error rates were controlled in both tests despite model misspecification. The TME test was more powerful than the CME test when the two mediation effects are in the same direction, while the CME test was more powerful than the TME test when the two mediation effects are in opposite direction. Multiple mediator analysis had increased power to detect mediated trans-eQTLs, especially in large samples. In the HapMap3 data, we identified 11 mediated trans-eQTLs that were not detected by the single mediator analysis in the combined samples of African populations. Moreover, the mediated trans-eQTLs in the HapMap3 samples are more likely to be trait-associated SNPs. In terms of computation, although there is no limit in the number of mediators in our model, analysis takes more time when adding additional mediators. In the analysis of the HapMap3 samples, we included at most 5 cis-gene mediators. Majority of the trios we considered have one or two mediators.

**Conclusions:**

Trans-eQTLs are more likely to associate with multiple cis-genes than randomly selected SNPs. Mediation analysis with multiple mediators improves power of identification of mediated trans-eQTLs, especially in large samples.

**Electronic supplementary material:**

The online version of this article (10.1186/s12859-019-2651-6) contains supplementary material, which is available to authorized users.

## Background

**Fig. 1:**
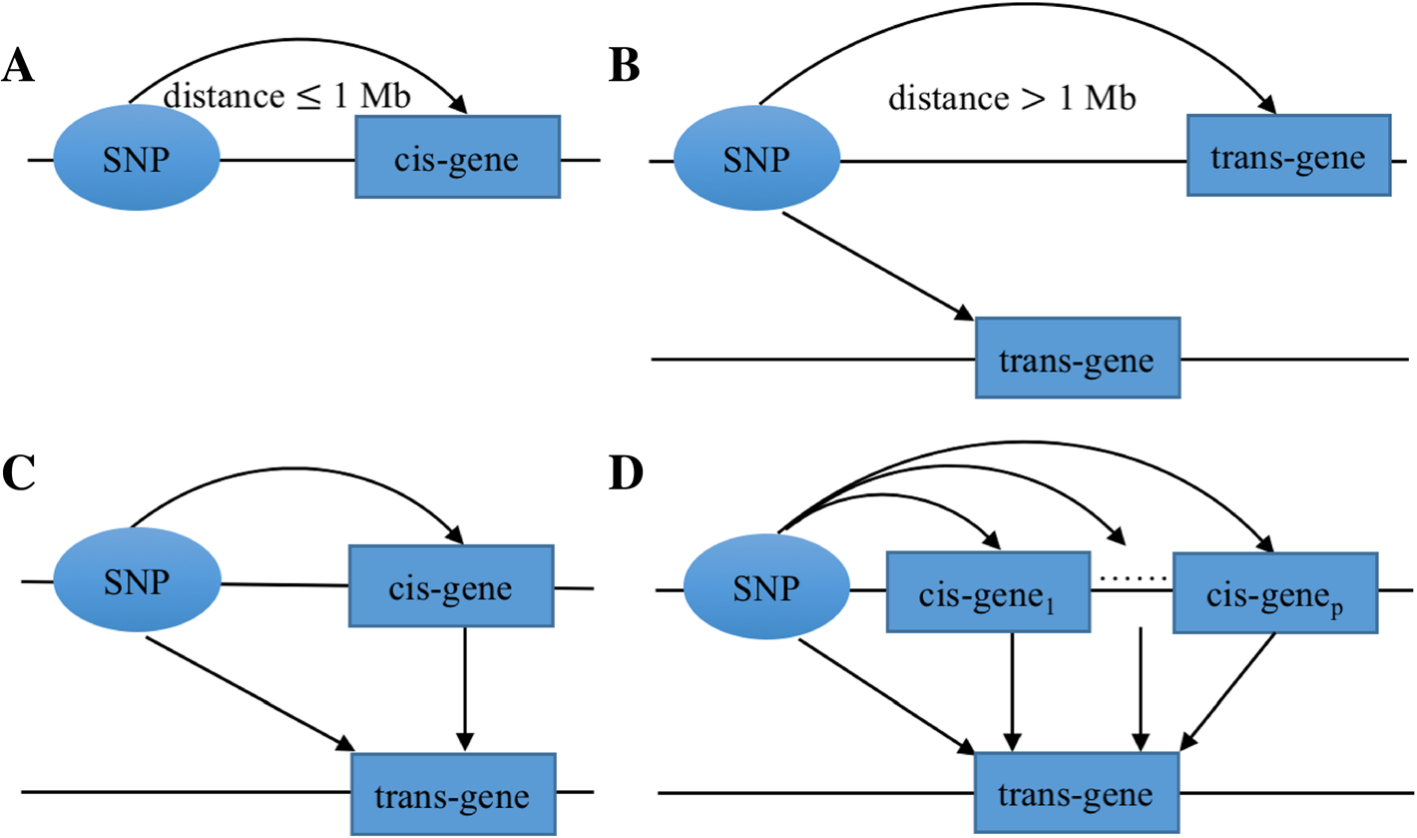
Graphical representation of eQTLs. **a** cis-eQTL, **b** trans-eQTL, **c** mediated trans-eQTL with a single cis-mediator, and **d** mediated trans-eQTL with multiple cis-mediators

Expression quantitative trait loci (eQTLs) are genetic variants that influence expression levels of mRNA transcripts. Cis-eQTLs commonly refer to genetic variations that act on local genes (Fig. 1a), and trans-eQTLs are those that act on distant genes and genes residing on different chromosomes (Fig. 1b). Identification of eQTLs can help advance our understanding of genetics and regulatory mechanisms of gene expression in various organisms [1]. Consistent findings suggest that many genes are regulated by nearby single nucleotide polymorphisms (SNPs), and the identified cis-eQTLs are typically close to transcription start sites. In contrast to cis-eQTLs, trans-eQTL identification is much more challenging because a greater number of SNP-gene pairs are tested for trans-association. In order to achieve the same power, analysis of trans-eQTLs requires a much larger sample size and/or effect than that in the cis-eQTL analysis. However, trans-eQTLs tend to have weaker effects than cis-eQTLs [2]. Several methods have been developed to improve trans-eQTL detection, such as reducing the multiple-testing burden based on pairwise partial correlations from the gene expression data to increase power [3], and constructing or selecting variables to control for unmeasured confounders that may lead to spurious association [4–6].

Moreover, the biological mechanisms underlying trans-eQTLs are less understood. Previous studies have shown that trans-eQTLs are more likely to be cis-eQTLs than randomly selected SNPs in the human genome [2, 7], suggesting that trans-eQTLs may regulate expression of remote genes by altering the expression of nearby genes. Recently, mediation analysis has become a popular tool to explore trans-association mediated by cis-regulators [2, 6, 8]. These studies used mediation test assuming a single mediator (Fig. 1c). However, gene expression levels are not independent due to the complex regulatory mechanisms. Correlation between genes may violate the assumptions that are required to identify mediation effects if other cis-genes also affect the trans-gene in study. Mediation analysis with multiple mediators has been applied in genomics [9–11], epigenetics [12], and epidemiological studies [13]. Mediation with two mediators was used in [9, 10, 13] and mediation with high dimensional mediators was implemented in [11, 12].

In this paper, we showed that the assumptions in the multivariate extension of mediation analysis are more likely to be satisfied than that in the single-mediator model (Additional file 1). We also found that trans-eQTLs are more likely to associate with more than one cis-gene than randomly selected SNPs in various tissues from the GTEx database. Then, we developed a computational method to identify trans-eQTLs that are mediated by multiple mediators (Fig. 1d). In simulation studies, we demonstrated that the multiple mediator approach increases the statistical power of identification of mediated trans-eQTLs. The improvement is more pronounced in large sample size. We applied the method to the HapMap3 dataset and identified 11 mediated trans-eQTLs that were not detected by the single mediator analysis in the combined samples of African populations. Lastly, we illustrated that mediated trans-eQTLs are more likely to be trait-associated SNPs in genome-wide association studies (GWAS). These findings advance our knowledge of gene regulation.

## Methods

### Dataset description

Genotype and gene expression data were retrieved from six HapMap3 populations, LWK (Luhya in Webuye, Kenya), MKK (Maasai in Kinyawa, Kenya), YRI (Yoruba in Ibadan, Nigeria), CEU (Utah residents with Northern and Western European ancestry from the CEPH collection), CHB (Han Chinese in Beijing, China), and JPT (Japanese in Tokyo, Japan) [14]. There are 83, 135, 107, 107, 79, and 81 individuals in each population, respectively. The greater genetic diversity in African populations (LWK, MKK, YRI) tends to increase the power of eQTL detection [15]. Therefore, we performed analyses in the three populations separately and in the combined samples. Due to the sample size below 100 in CHB and JPT, we combined the two populations into a sample of Asian populations. Processed expression data profiling on the Illumina Human-6 v2 Expression BeadChip array for the HapMap3 samples were downloaded from ArrayExpress (accession numbers E-MTAB-264 and E-MTAB-198). We also downloaded the V6p release from the GTEx database, which provides a complete list of cis- and trans-eQTLs identified in the GTEx study [16].

### Genotype data processing

In the quality control step, a series of filters were applied to remove samples and SNPs with poor quality in each population. We removed samples with a call rate less than 0.97; next we retained autosomal SNPs with a missing rate less than 0.08 and minor allele frequency (MAF) greater than 0.10; finally, SNPs that failed the Hardy-Weinberg test (*p*-value <10^-5^) were removed. We then converted the SNP coordinates according to the human reference genome hg38. In addition, we found that some SNPs were in complete Linkage Disequilibrium (LD) with each other or mapped to identical genome positions. For such cases, we randomly selected one SNP to be included in the analysis. The number of individuals and SNPs before and after quality control were listed in Table 1. In total, there are 740,158 SNPs retained in the combined samples of African populations and 540,684 SNPs in the combined samples of Asian populations.

**Table 1:** The number of individuals and SNPs before and after quality control in the HapMap3 data

	Sample Size	# SNPs before quality control	# SNPs after quality control
LWK	83	1533540	953834
MKK	135	1541375	989807
YRI	107	1505108	943161
LWK+MKK+YRI	325	NA^a^	740158
CEU	107	1416121	787357
CHB	79	1332120	675811
JPT	81	1300764	643419
CHB+JPT	160	NA	540684

^a^*NA*: data is not available

### Gene expression data processing

There are 21,800 probes in the microarray gene expression in the HapMap3 samples. Among them, 20,439 probes were mapped to the reference genome. We then removed probes that were mapped to multiple genes or non-autosomes, resulting in 19,832 probes corresponding to 19,643 unique genes. We further removed probes with low variance or low intensity and performed quantile normalization to reduce inter-individual variation [17]. Mediation analysis was applied to the probe level data, mainly because multiple probes in a gene represent different isoforms of this gene and merging them may lose information. More importantly, probes mapped to the same gene were weakly correlated in the HapMap3 data.

### Population stratification and confounders in gene expression data

In the single population analysis (LWK, MKK, YRI, CEU), we adopted the strategy of [18] to correct for population admixture in LWK and MKK. We used the EIGENSTRAT program [19] to select the top 10 principal components (PCs) generated from the SNP genotype data as covariates. In the combined samples of African populations and Asian populations, 20 PCs from the genotype data were included in the analysis. To adjust for batch effects and unmeasured confounders in the gene expression datasets, we used the probabilistic estimation of expression residuals (PEER) method [20]. Following the GTEx analysis [21], the number of factors for PEER was determined by the sample size. We included 15 factors for datasets with less than 150 samples, 30 factors for datasets with sample size between 150 and 250, and 35 factors for datasets with more than 250 samples. Gender was also included as a covariate in all analyses.

### eQTL analysis

We conducted genome-wide eQTL analysis using the R package, Matrix eQTL [22]. SNPs and probes within 1 Mb were tested for cis-association. All inter-chromosomal SNP-probe pairs as well as intra-chromosomal SNP-probe pairs that are more than 1 Mb apart were tested for trans-association.

### Enrichment analysis

The motivation of our work was based on the observation that many trans-eQTLs are also identified as cis-eQTLs and they are often associated with more than one cis-gene in the GTEx database. In order to test whether the association with multiple cis-genes is over-represented in trans-eQTLs, we compared the proportion of trans-eQTLs that are associated with more than one cis-gene with that in the human genome. We considered the trans-eQTLs reported in the GTEx V6p dataset and those identified in the HapMap3 dataset. Permutation tests were used to assess significance. To elaborate, for the trans-eQTLs reported in the GTEx V6p dataset, we randomly sampled the same number of SNPs with matched MAF from the 1000 Genomes Project [23] and calculated the proportion of SNPs that are associated with multiple cis-genes. The empirical *p*-value was obtained by resampling 1000 times. The same test procedure was applied to the trans-eQTLs identified in the HapMap3 dataset.

To understand the role of mediated trans-eQTLs in disease association, we performed Fisher’s exact test to assess the enrichment of trait-associated SNPs in the trans-eQTLs identified by our method. The trait-associated SNPs were obtained from the NHGRI GWAS catalog [24].

### Mediation analysis

To identify trans-eQTLs that are mediated by one or more cis-genes, we first selected candidate trios, composed of SNP, one or multiple cis-genes, and trans-gene. The trios were selected based on the following criteria. First, trans SNP-gene pairs were selected if their *p*-value is less than 10^-6^. The *p*-value cutoff was chosen to reduce the multiple-testing burden [6]. Second, cis-genes that are associated with the SNPs identified from the first step at a genome-wide false discovery rate (FDR) less than 0.05 were selected as candidate cis-mediators.

In all the analyses described below, we assume gene expression data have been normalized and transformed so that the expression values approximately follow a normal distribution. In mediation analysis with a single mediator, we followed the test procedure in [25]. The bootstrap *p*-value was used to assess significance when testing the single mediation effect (SME). In mediation analysis with multiple mediators, we considered the following model. For the *i*^*th*^ subject, let *Y*_*i*_ be the expression level of a trans-gene, *X*_*i*_ be the SNP genotype coded by the number of minor alleles, ***M***_*i*_ = (*M*_*i*1_, ⋯⋯, *M*_*ip*_)^*T*^be the expression levels of the *p* cis-genes, ***C***_*i*_ = (*C*_*i*1_, ⋯⋯, *C*_*iq*_)^*T*^be the *q* covariates. The mediation model is stated below:

$$ {Y}_i={\beta}_0+{X}_i{\beta}_X+{\boldsymbol{M}}_i^T{\boldsymbol{\beta}}_M+{\boldsymbol{C}}_i^T{\boldsymbol{\beta}}_C+{\varepsilon}_{Y_i} $$

1 $$ {M}_{ij}={\alpha}_{0j}+{X}_i{\alpha}_{Xj}+{\boldsymbol{C}}_i^T{\boldsymbol{\alpha}}_{Cj}+{\varepsilon}_{M_{ij}} $$

where ${\boldsymbol{\beta}}_M={\left({\beta}_{M_1},\cdots \cdots, {\beta}_{M_p}\right)}^T$is the effect of the *p* cis-genes on the trans-gene adjusting for the SNP and covariates, ***α***_*X*_ = (*α*_*X*1_, ⋯⋯, *α*_*Xp*_)^*T*^ is the effect of the SNP on the *p* cis-genes adjusting for covariates. ${\varepsilon}_{Y_i}$ and ${\varepsilon}_{M_{ij}}$ are measurement errors on gene expression. Here we assume ${\varepsilon}_{Y_i}\sim N\left(0,{\sigma}^2\right)$, ${\boldsymbol{\varepsilon}}_{M_i}={\left({\varepsilon}_{M_{i1}},\cdots \cdots, {\varepsilon}_{M_{ip}}\right)}^T\sim {N}_p\left(\mathbf{0},\boldsymbol{\Sigma} \right)$, and ${\varepsilon}_{Y_i}$ and ${\varepsilon}_{M_{ij}}$ are independent, but we allow dependence among cis-genes, i.e., the off-diagonal elements in the covariance matrix **Σ** can be non-zero [11].

Denote the total mediation effect (TME) as $\Delta  ={\boldsymbol{\alpha}}_X^T{\boldsymbol{\beta}}_M$ and the component-wise mediation effects (CME) as ***δ*** = (*δ*_1_, ⋯⋯, *δ*_*p*_)^*T*^, where ${\delta}_j={\alpha}_{Xj}{\beta}_{M_j}$ [11]. In the following, we focus on the hypothesis tests of TME and CME:

2 $$ {H}_0:\Delta  =0 $$

3 $$ {H}_0:\boldsymbol{\delta} =\mathbf{0} $$

where (2) consists of a broader class of null than (3). For example, when *δ*_*j*_ ’s are nonzero in different directions and sum to 0, the TME is zero while the CME is not. Thus, the CME test is of particular interest in the presence of the cancellation effect, which is evident in the HapMap3 dataset. That is, if a SNP has a positive mediation effect through one cis-gene and a negative mediation effect through another cis-gene, the CME test can be more powerful than the TME test, as demonstrated in the simulation studies.

Conventional multivariate tests for CME, such as likelihood ratio test, have limited power when there are a large number of mediators [26]. In our problem, we were less concerned because there are 1 or 2 cis-mediators in majority of the trios (see results in Additional file 2, Additional file 3, Additional file 4, Additional file 5, Additional file 6 and Additional file 7). We used the bootstrap method to assess significance. For comparison, we also tested the SME for each mediator in the trios that have multiple mediators, and the mediation effect was considered to be significant if at least one of the SME tests is significant.

### Simulation setup

We conducted simulation studies to evaluate the impact of model misspecification on type I error and statistical power. In detail, we considered three types of model misspecification: Scenario I, the true model has only one mediator while the analysis includes the true mediator and another irrelevant variable as the mediators; Scenario II, the true model has two mediators and the mediation effects are in the same direction; Scenario III, the true model has two mediators and the mediation effects are in opposite direction. In all three scenarios, the performance of the TME, CME, and SME tests are evaluated and compared. We considered sample size of 100 and 300 to mimic the sample size in the HapMap3 single population analysis and combined analysis.

Scenario I: The MAF of the SNP is set to 0.3. For the cis-regulatory effect in model (1), *α*_*X*1_ varies from 0.2 to 1, *α*_*X*2_ is fixed at 0.6, and${\alpha}_{01}={\alpha}_{02}={\beta}_0=0.5,{\beta}_{M_2}=0,{\beta}_X=0.3$. We assume an exchangeable covariance structure for ***ε***_*M*_ with the variance being 1 and the correlation coefficient being 0.2, and *ε*_*Y*_ follows the standard normal distribution. We set ${\beta}_{M_1}=0$ in the type I error experiments and ${\beta}_{M_1}=0.1$ in the power evaluation. The parameters are chosen to mimic the effects estimated in the HapMap3 dataset.

Scenario II: We set${\beta}_{M_2}=0$ and 0.1 to evaluate the type I error and the power respectively. The other parameters are set the same as in Scenario I.

Scenario III: We set$\kern0.50em {\beta}_{M_2}=0$ and −0.1 to evaluate the type I error and the power respectively. The other parameters are set the same as in Scenario I.

## Results

### Trans-eQTLs are more likely to associate with multiple cis-genes

Previous studies showed that trans-eQTLs are more likely to associate with cis-genes [2, 7], which lays the foundation for the employment of mediation analysis in trans-eQTL studies. To justify multiple mediators, we hypothesized that trans-eQTLs tend to associate with more than one cis-gene, and validated this hypothesis in the GTEx dataset. In 14 out of the 22 tissues available in the GTEx database, trans-eQTLs were found to be significantly associated with two or more cis-genes, and the sample sizes are all greater than 100 (Table 2). In the remaining 8 tissues, sample size is less than 100 in 4 tissues, and no more than 3 trans-associations were observed in 5 tissues. Consistent with the GTEx dataset, we also observed an enrichment of multiple cis-genes in trans-eQTLs in MKK, YRI, CEU, and the combined samples of African populations and Asian populations in the HapMap3 dataset (Table 3). The only exception is the LWK population, possibly because the power of identifying cis- and trans-eQTLs is limited at the sample size of 83. Thus, multiple mediators are prevalent among trans-eQTLs. In the upcoming sections, we developed and evaluated statistical tests to identify trans-eQTLs in a multiple-mediator setup, and then applied the method in the HapMap3 dataset.

**Table 2:** Enrichment results in different tissues in the GTEx database

Tissue	Sample size	Cis-association (FDR<0.05)	Trans-association (FDR<0.1)	Trans-association^a^	Trans-association^b^	Empirical *p*-value
Adipose subcutaneous	298	1282841	45	10	10	0.009
Adrenal gland	126	396098	1	0	0	1
Artery aorta	197	853794	288	2	243	<0.001
Artery tibial	256	1210709	12	3	1	0.156
Brain hypothalamus	81	150415	2	0	0	1
Brain nucleus accumbens (basal ganglia)	93	244929	2	0	0	1
Brain putamen (basal ganglia)	82	183240	11	0	0	1
Cells transformed fibroblasts	272	1283340	658	19	376	<0.001
Colon transverse	169	581854	18	0	6	0.006
Esophagus mucosa	241	1089061	980	122	145	<0.001
Esophagus muscularis	218	997653	15	5	10	0.001
Heart left ventricle	190	605253	3	3	0	1
Lung	278	1068860	98	0	8	0.041
Muscle skeletal	361	1100532	59	0	35	<0.001
Nerve tibial	256	1454889	30	10	1	0.288
Pancreas	149	515665	283	0	244	<0.001
Prostate	87	177994	1	0	0	1
Skin not sun-exposed (suprapubic)	196	722868	11	0	6	0.004
Skin sun-exposed (lower leg)	302	1306762	64	13	25	<0.001
Testis	157	1121727	203	34	68	0.013
Thyroid	278	1551668	2120	230	1390	<0.001
Whole blood	338	1036239	35	2	24	<0.001

^a^Trans-association in which the trans-eQTL is also associated with 1 cis-gene

^b^Trans-association in which the trans-eQTL is also associated with 2 or more cis-genes

**Table 3:** Enrichment results in the HapMap3 data

Population	Sample size	Cis-association (FDR<0.05)	Trans-association (FDR<0.1)	Trans-association^a^	Trans-association^b^	Empirical *p*-value
LWK	83	6838	7	1	0	1
MKK	135	17889	46	6	3	<0.001
YRI	107	18239	51	18	10	<0.001
LWK+MKK+YRI	325	56437	192	35	64	<0.001
CEU	107	26506	210	18	60	<0.001
CHB+JPT	160	42953	135	56	29	<0.001

^a^Trans-association in which the trans-eQTL is also associated with 1 cis-gene

^b^Trans-association in which the trans-eQTL is also associated with 2 or more cis-genes

### Simulation studies

In simulations, we studied the effect of model misspecification in three scenarios of trans-eQTL identification (see Simulation setup in Methods) from two perspectives, the type I error and the statistical power, and we compared three tests, TME, CME, and SME.

**Fig. 2:**
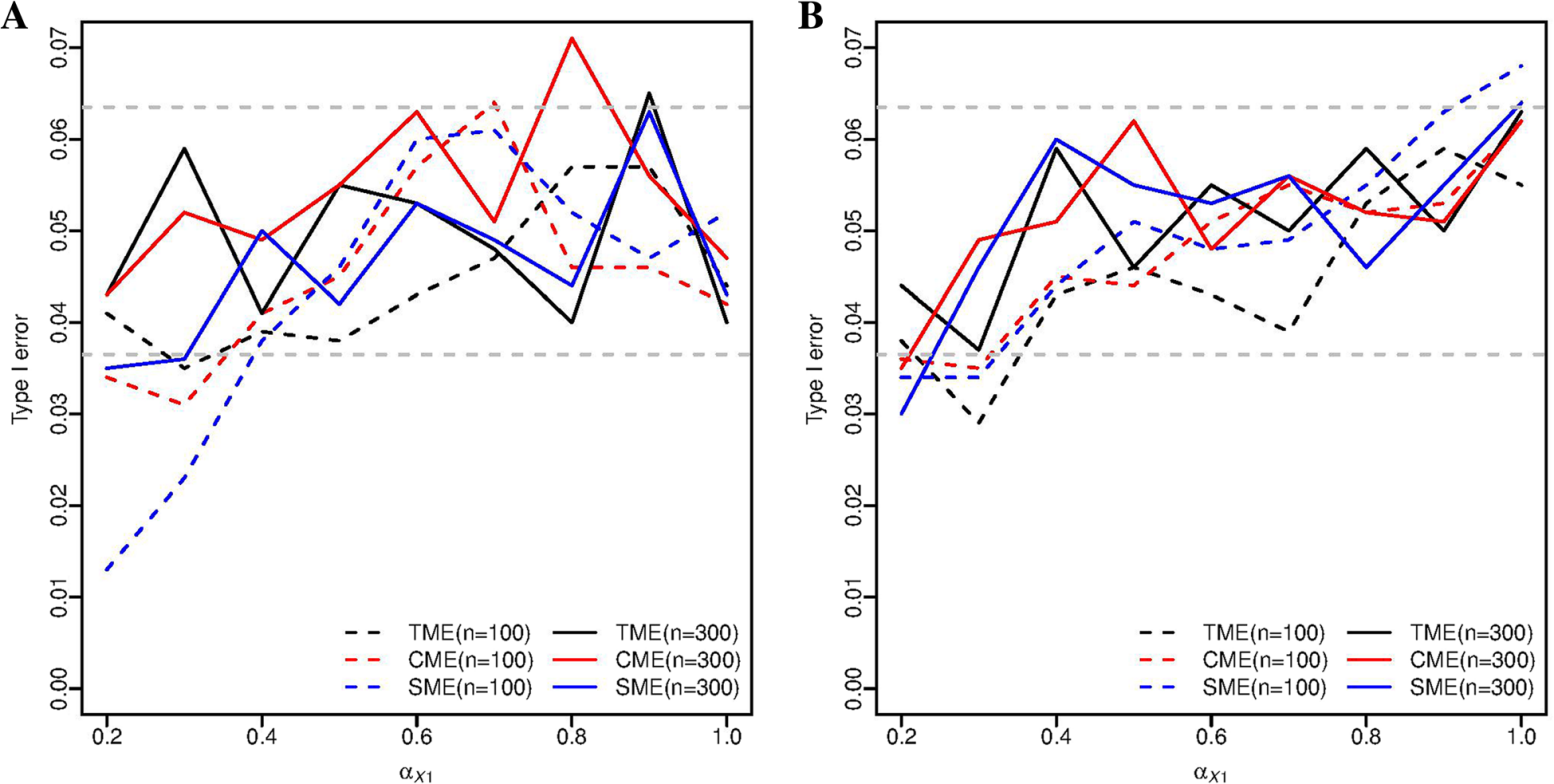
Empirical type I error of the TME, CME, and SME tests, based on 1,000 simulation replicates, α=0.05. **a** type I error in Scenario I, and **b** type I error in Scenario II/III. The two horizontal grey dashed lines are the 95% confidence interval (0.0365-0.0635)

**Fig. 3:**
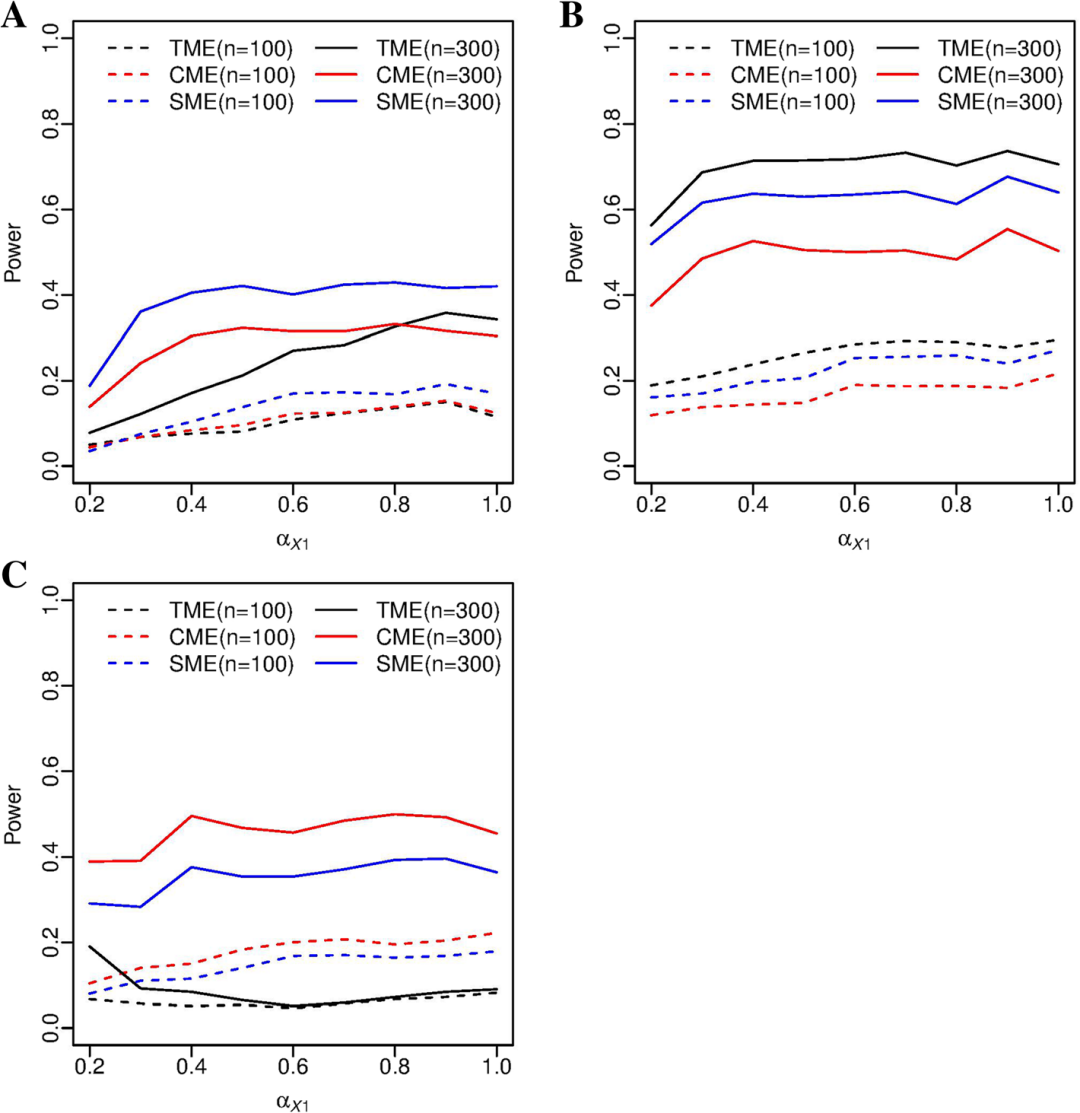
Empirical power of the TME, CME, and SME tests, based on 1,000 simulation replicates, α=0.05. **a** power in Scenario I, **b** power in Scenario II, and **c** power in Scenario III

In Scenario I, the type I error rates did not differ significantly from the nominal level of 0.05 even though a second mediator was falsely included in the analysis (Fig. 2a), and the results were consistent in all three tests we considered. In terms of power, as we expected, the SME test (true model) achieved the highest power, while the TME and CME tests had reduced power due to falsely including a cis-gene that does not mediate the trans-association (Fig. 3a). However, the power difference between SME and TME quickly diminishes as the mediated effect of the true mediator increases. In the mediated trans-eQTL problem, we pre-select trios for mediation test, and there is no guarantee that false mediators are excluded at this step. However, as shown in simulations, the type I error was under control, at the expense of power loss.

In Scenarios II and III, the null hypotheses are identical for each test respectively, thus we presented the type I error results in one graph (Fig. 2b). We can see that the type I error was under control when either one or both mediators are included in the model. In terms of power, the TME test was more powerful than the CME test in Scenario II when the two mediation effects are in the same direction. In contrast, the TME test was less powerful than the CME test in Scenario III when the two mediation effects are in opposite direction. The SME test lost power due to leaving out one of the two cis-mediators, and its power fell between that of TME and CME (Fig. 3b, c). It is noteworthy that for the SME test, we actually performed two separate tests for each of the mediators, and the rejection of either one leads to the final rejection. The results were consistent with [11], where similar directionality effect was reported. The power difference of the three tests increases as the sample size increases. When the mediation effects were in different direction, the power of the TME test declined until *α*_*X*1_ reached 0.6 when the two mediation effects were cancelled. After that, the power of the TME test rose with the value of *α*_*X*1_, but was still inferior to that of CME and SME (Fig. 3c). In summary, the single mediator model loses power when multiple mediators are present, and the optimal choice of the hypothesis test depends on the unknown directionality of the mediation pathways.

### Identification of mediated trans-eQTLs: application to the HapMap3 dataset

We applied the mediation tests to LWK, MKK, YRI, CEU, and the combined samples of African populations and Asian populations in the HapMap3 dataset. In each population, mediated trans-eQTLs with *p*-values less than 0.05 are shown in Table 4 (more details in Additional file 2, Additional file 3, Additional file 4, Additional file 5, Additional file 6 and Additional file 7). The three tests gave similar results in the single population analysis, perhaps due to the small sample size. In the combined samples of African populations, 291 (24.3%) trans-eQTLs were associated with two or more cis-genes. Among the 248 trans-eQTLs associated with two cis-genes, 70 trios were identified by both the TME and CME tests, 13 trios in which the estimated mediation effects were in the same direction were identified by the TME test but not the CME test, and 17 trios in which the estimated mediation effects were in opposite direction were identified by the CME test but not the TME test. All the 89 trios detected by the SME test were also identified by either the TME or CME test. In total, we identified 11 mediated trans-eQTLs that were not detected by the single mediator analysis (Table 5). In the Asian populations, 254 (26.1%) trans-eQTLs were associated with two or more cis-genes. Among the 195 trans-eQTLs associated with two cis-genes, 33 trios were identified by both the TME and CME tests, 12 trios in which the estimated mediation effects were in the same direction were identified by the TME test but not the CME test, 2 trios were identified by the CME test but not the TME test, and 4 trios in which the estimated mediation effects were in opposite direction were identified by the CME test but not the TME test. All the 45 trios detected by the SME test were also identified by either the TME or CME test. There are 6 mediated trans-eQTLs that were not detected by the single mediator analysis. Similar results were obtained when the trans-eQTL *p*-value threshold was set at 10^-7^ (data not shown).

**Table 4:** Mediated trans-eQTLs with *p*-value <0.05 in the HapMap3 data

Population	Sample Size	# cis-mediators	# trios tested	# trios with *p*-value<0.05			
				TME	CME	TME+CME	SME
LWK	83	1	139	NA^a^	NA	NA	8
		2	26	5	3	5	4
MKK	135	1	426	NA	NA	NA	54
		2	32	4	6	6	7
		4	2	0	0	0	0
YRI	107	1	463	NA	NA	NA	43
		2	47	10	11	11	11
		3	8	5	5	5	5
LWK+MKK+YRI	325	1	905	NA	NA	NA	77
		2	248	83	87	100	89
		3	35	6	11	11	11
		4	7	1	2	2	2
		5	1	1	1	1	1
CEU	107	1	527	NA	NA	NA	60
		2	215	57	61	75	73
		3	14	1	3	3	2
CHB+JPT	160	1	721	NA	NA	NA	64
		2	195	45	39	51	45
		3	41	4	3	4	3
		4	13	1	1	1	1
		5	5	0	0	0	0

^a^*NA*: the tests are not applicable

**Table 5:** Mediated trans-eQTLs that were detected by the multiple mediator analysis but not detected by the single mediator analysis in the combined samples of African populations

SNP	Chr (SNP)	Position	Cis-gene_1_	Cis-gene_2_	Chr (trans-gene)	Trans-gene
rs2024679	6	29259340	*ZKSCAN3*	*PGBD1*	17	*NCOR1*
rs3117327	6	29271373	*ZKSCAN3*	*PGBD1*	17	*NCOR1*
rs3135392	6	32441465	*HLA-DRB5*	*HLA-DRB1*	4	*RPL34*
rs2239804	6	32443746	*HLA-DRB5*	*HLA-DRB1*	4	*RPL34*
rs9270623	6	32597554	*HLA-DRB5*	*HLA-DRB1*	4	*RPL34*
rs642093	6	32614298	*HLA-DRB5*	*HLA-DRB1*	4	*RPL34*
rs2097431	6	32623056	*HLA-DRB5*	*HLA-DRB1*	12	*ATP5MFP5*
rs9272105	6	32632222	*HLA-DRB5*	*HLA-DRB1*	4	*RPL34*
rs10987642	9	127411687	*SLC2A8*	*ZNF79*	17	*RPL12P38*
rs10511793	9	26924623	*CAAP1*	*IFT74*	7	*BRI3*
rs2835187	21	35967194	*SETD4*	*CBR1*	3	*PCOLCE2*

### Replication of trans-eQTLs and mediated trans-eQTLs

We demonstrated the replication of trans-eQTLs from LWK, MKK, YRI, and the combined samples of African populations. When the FDR was controlled at 0.1, the trans-eQTLs identified in LWK, MKK, YRI, and the combined samples have a large overlap (Additional file 8). Among the 7 trans-eQTLs identified in LWK, all of them were also identified in another population or the combined samples. Among the 46 trans-eQTLs identified in MKK, 23 of them were also identified in another population or the combined samples. Among the 51 trans-eQTLs identified in YRI, 30 of them were also identified in another population or the combined samples. Additionally, we compared the results with that from a previous study in which the FDR of trans-eQTLs was set at 0.05 [2]. There were 2, 5, 5, and 20 trans-eQTLs identified by our method in LWK, MKK, YRI, and the combined samples respectively that were previously reported (more details in Additional file 9). The relatively low rates of replication with the previous study may be explained by genetic and environmental differences between populations [2]. Next, we evaluated the replication of mediated trans-eQTLs across populations (Additional file 10). 7 of 13 mediated trans-eQTLs identified in LWK were also identified in another population or the combined samples. 13 of 61 mediated trans-eQTLs identified in MKK were also identified in another population or the combined samples. 10 of 59 mediated trans-eQTLs identified in YRI were also identified in another population or the combined samples. For those trans-eQTLs that have inconsistent mediation across populations, it may be due to different gene regulatory mechanisms between populations [27]. Lastly, we observed that trait-associated SNPs are enriched in the mediated trans-eQTLs identified in the combined samples of African populations and Asian populations (Table 6).

**Table 6:** Enrichment results of trait-associated SNPs in the mediated trans-eQTLs identified in the HapMap3 data

Population	Mediated trans-eQTL	Trait association		Enrichment *p*-value
		Yes	No	
LWK	Yes	2	11	0.013
	No	13180	940641	
MKK	Yes	3	56	0.055
	No	14365	975383	
YRI	Yes	2	53	0.172
	No	12836	930270	
LWK+MKK+YRI	Yes	18	155	1.484 × 10^−10^
	No	10901	729084	
CEU	Yes	24	106	<2.2 × 10^−16^
	No	13403	773824	
CHB+JPT	Yes	15	98	1.019 × 10^−9^
	No	9144	531427	

### Examples of mediated trans-eQTLs

**Fig. 4:**
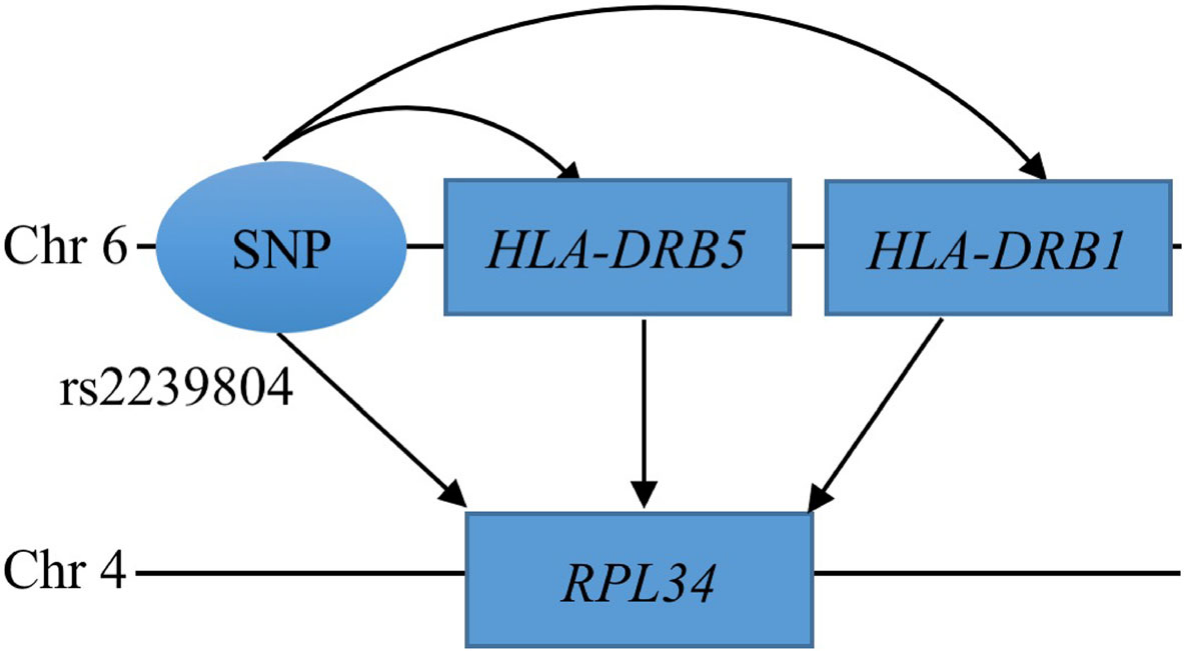
Mediation diagram of the trans-association between rs2239804 and *RPL34*

The identified trans-eQTLs that are mediated by multiple mediators may bring new biological insight in gene regulation. For example, the *RPL34* gene on chromosome 4 was found to be trans-associated with 5 SNPs on chromosome 6 through the mediation of *HLA-DRB5* and *HLA-DRB1* (Table 5). *RPL34* was previously reported to be trans-associated with the SNP rs2395185 in human monocytes [28], and the association was found unique to TLR4 activation, which plays a key role in innate immunity [29]. However, the biological mechanism underlying this trans-association is unknown. Our study identified the mediated trans-association of *RPL34* and the SNP rs2239804 which is in LD with rs2395185 (*r*^2^ = 0.53), suggesting a mediating pathway of the previously reported trans-regulation of *RPL34* (Fig. 4). The SNP rs2395185 and the two cis-mediators, *HLA-DRB5* and *HLA-DRB1*, were reported to be susceptible to ulcerative colitis [30], and the two HLA genes were also identified in the rheumatoid arthritis GWAS [31]. The dysfunction of innate immunity is critically important in the pathogenesis of ulcerative colitis [32] and rheumatoid arthritis [33]. Thus, the identified mediated trans-eQTLs not only suggest a biological mechanism for the trans-association of rs2239804 and *RPL34*, but also suggest a role of the mediated pathway in the disease etiology of ulcerative colitis and rheumatoid arthritis.

## Discussion

eQTL studies have shed enormous light on gene regulatory mechanisms. Significant progress has been made to integrate eQTL information with genome-wide association signals to explain SNP-phenotype associations and prioritize genes and variants for functional studies [34–36]. The ongoing efforts such as GTEx and the HapMap Project have greatly expanded current knowledge of eQTLs. However, the identification and interpretation of trans-eQTLs remain a challenging yet important topic. In this work, we developed a computational method to identify trans-eQTLs that are mediated by multiple mediators, and demonstrated its superiority to the single mediator test in mediation analysis.

Previous studies considered the identification of cis-transcripts that mediate the effects of trans-eQTLs on distant genes in a single-mediator setting [2, 6, 8], and may be subject to potential model misspecification. One innovative aspect of our work is to employ the multiple-mediator analysis to identify mediated trans-eQTLs. We observed that the associations of trans-eQTLs with more than one cis-gene are prevalent in the GTEx and HapMap3 datasets. Thus, mediation analysis allowing for multiple mediators would be less sensitive to model misspecification, and as a result improve the statistical power of the tests. Applied to the HapMap3 data, our approach allowing for multiple mediators identified 11 mediated trans-eQTLs that were not detected in the single mediator analysis.

There are several caveats in our work. First, unmeasured confounders may not be fully accounted for in the mediation analysis due to the biological complexity in gene regulatory networks. The influence of potential confounders was further evaluated in the single-mediator setting [37]. Sensitivity analysis in the mediation with multiple mediators will be investigated in future studies. Second, we cannot make causal claims based on the detected mediation effects because the observed mediations simply explain trans-associations but not establish causal relationships. Third, the selection of cis-gene mediators is completely data-driven in the current study. It would be of great interest to integrate the knowledge of gene networks into the mediation framework.

## Conclusions

We implemented a multiple-mediator analysis approach to identify mediated trans-eQTLs. In simulation studies, we illustrated that our method improves the statistical power of identification of mediated trans-eQTLs compared to the single mediator analysis. Furthermore, we identified 11 mediated trans-eQTLs that were not detected by the single mediator analysis in the HapMap3 data.

## Additional files

Additional file 1: Assumptions in mediation analysis. This document explaining that the assumptions in the multivariate extension of mediation analysis are more likely to be satisfied than that in the single-mediator model. (DOCX 14 kb)

Additional file 2: Mediated trans-eQTLs in LWK. (XLS 64 kb)

Additional file 3: Mediated trans-eQTLs in MKK. (XLS 73 kb)

Additional file 4: Mediated trans-eQTLs in YRI. (XLS 71 kb)

Additional file 5: Mediated trans-eQTLs in the combined samples of African populations. (XLS 106 kb)

Additional file 6: Mediated trans-eQTLs in CEU. (XLS 94 kb)

Additional file 7: Mediated trans-eQTLs in the combined samples of Asian populations. (XLS 91 kb)

Additional file 8: Venn diagram of trans-eQTLs in African populations. (PNG 81 kb)

Additional file 9: Overlap of trans-eQTLs with a previous study [2]. (XLS 52 kb)

Additional file 10: Venn diagram of mediated trans-eQTLs in African populations. (PNG 82 kb)

## Abbreviations

CEU: Utah residents with Northern and Western European ancestry from the CEPH collection
CHB: Han Chinese in Beijing, China
CME: Component-wise mediation effect
eQTL: Expression quantitative trait locus
FDR: False discovery rate
GWAS: Genome-wide association study
JPT: Japanese in Tokyo, Japan
LD: Linkage disequilibrium
LWK: Luhya in Webuye, Kenya
MAF: Minor allele frequency
MKK: Maasai in Kinyawa, Kenya
PC: Principal component
PEER: Probabilistic estimation of expression residuals
SME: Single mediation effect
SNP: Single nucleotide polymorphism
TME: Total mediation effect
YRI: Yoruba in Ibadan, Nigeria
