# Supplementary material for: Identification of trans-eQTLs using mediation analysis with multiple mediators
Source: BMC Bioinformatics. 2019 Mar 29;20(Suppl 3):126. doi: 10.1186/s12859-019-2651-6 (PMC6440281; doi:10.1186/s12859-019-2651-6)
Supplement: Supplementary file 1 — Assumptions in mediation analysis. This document explaining that the assumptions in the multivariate extension of mediation analysis are more likely to be satisfied than that in the single-mediator model. (DOCX 14 kb) [file 12859_2019_2651_MOESM1_ESM.docx]

**Additional file 1:** Assumptions in mediation analysis

In mediation analysis, four assumptions are made: (1) no unmeasured exposure-outcome confounding, (2) no unmeasured mediator-outcome confounding conditional on the exposure, (3) no unmeasured exposure-mediator confounding, and (4) no unmeasured mediator-outcome confounder that is caused by the exposure [1-2].

Assumptions (1) and (3) is commonly satisfied with the randomization of the exposure [3]. In this work, the exposure is the SNP genotype, which can be treated as being randomized. Incorporating multiple mediators is less likely to violate Assumption (4) [4]. Similarly, including multiple mediators is more likely to satisfy Assumption (2) because many cis-genes associated with the same SNP are correlated.

**References**

1. Pearl J. Direct and indirect effects. In: Proceedings of the Seventeenth Conference on Uncertainty and Artificial Intelligence. San Francisco, CA: Morgan Kaufmann; 2001:411-20.
2. Vanderweele TJ. Bias formulas for sensitivity analysis for direct and indirect effects. Epidemiology. 2010;21:540-51.
3. Cox MG, Yasemin KS, Milica M, MacKinnon DP. Sensitivity plots for confounder bias in the single mediator model. Eval Rev. 2013;37:405-31.
4. VanderWeele TJ, Vansteelandt S. Mediation analysis with multiple mediators. Epidemiol Method. 2014;2:95-115.
