# Supplementary figures and images for: Identification of trans-eQTLs using mediation analysis with multiple mediators
Source: BMC Bioinformatics. 2019 Mar 29;20(Suppl 3):126. doi: 10.1186/s12859-019-2651-6 (PMC6440281; doi:10.1186/s12859-019-2651-6)

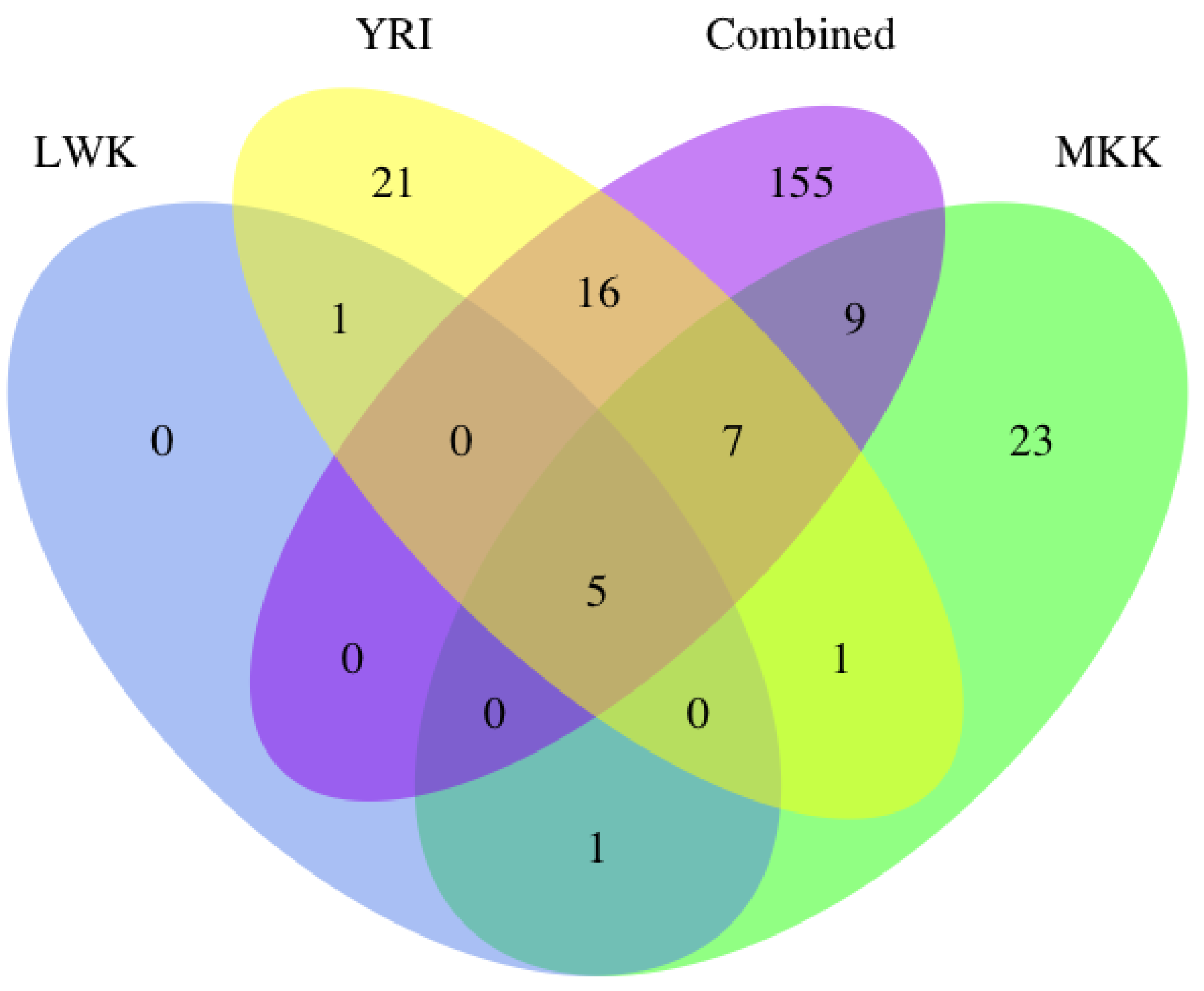

Supplement: Supplementary file 8 — Venn diagram of trans-eQTLs in African populations. (PNG 81 kb) [file 12859_2019_2651_MOESM8_ESM.png]

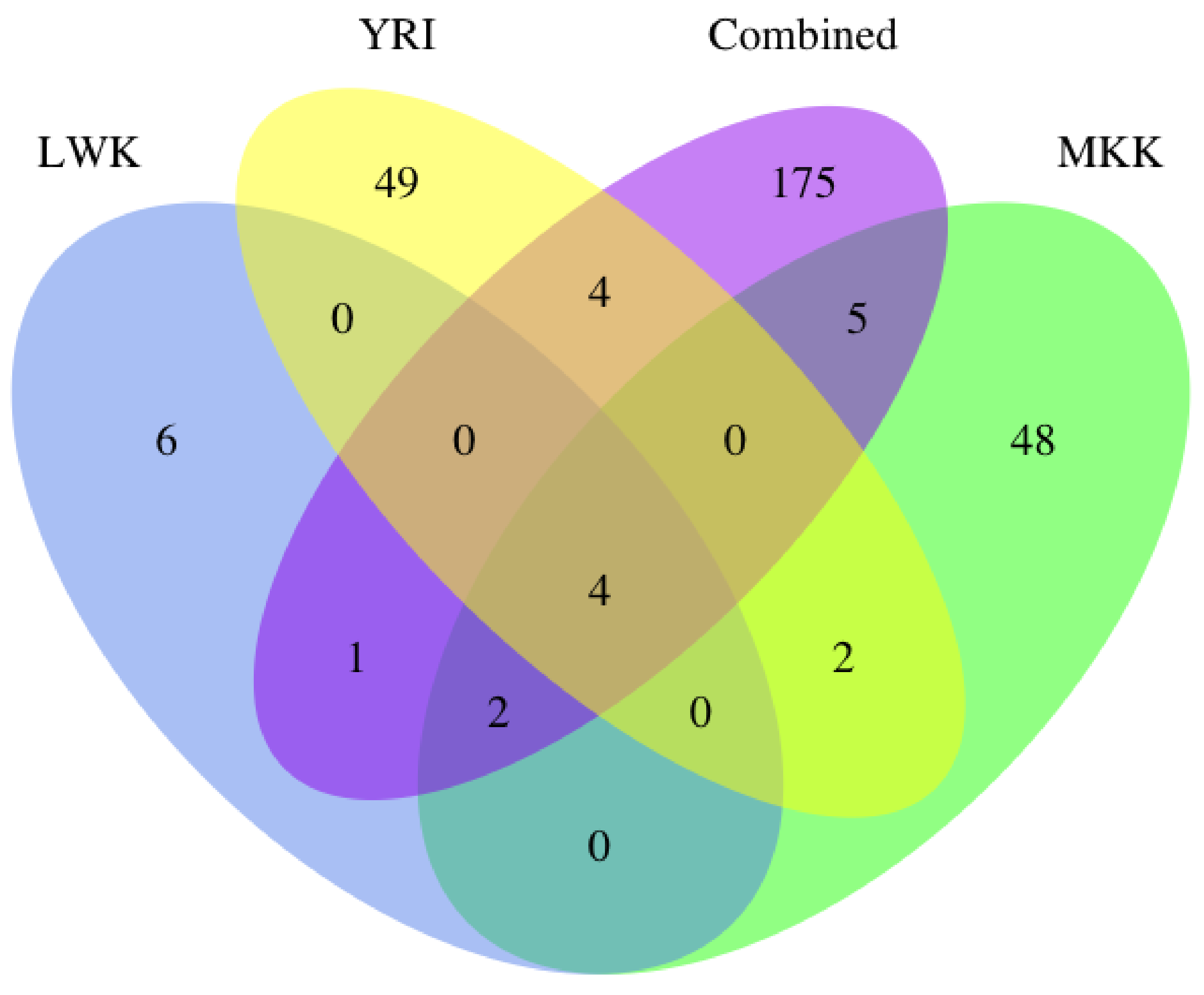

Supplement: Supplementary file 10 — Venn diagram of mediated trans-eQTLs in African populations. (PNG 82 kb) [file 12859_2019_2651_MOESM10_ESM.png]
